# Kinesiological Rehabilitation in a Young Adult with Biceps Femoralis Arteriovenous Malformation: A Case Study

**DOI:** 10.3390/jfmk9040225

**Published:** 2024-11-08

**Authors:** Giulia Di Martino, Carlo della Valle, Marco Centorbi, Paola Bovolenta, Giovanni Fiorilli, Giuseppe Calcagno, Alessandra di Cagno, Enzo Iuliano

**Affiliations:** 1 Department of Medicine and Health Sciences, University of Molise, 86100 Campobasso, Italy; giulia.dimartino21@gmail.com (G.D.M.); carlodv95@gmail.commarco.centorbi@hotmail.it (M.C.); fiorilli@unimol.it (G.F.); 2 Department of Neurosciences, Biomedicine and Movement, University of Verona, 37134 Verona, Italy; 3 Faculty of Psychology, eCampus University, 22060 Novedrate, Italy; bovolenta.paola@yahoo.it (P.B.); or enzo.iuliano84@gmail.com (E.I.); 4 Department of Movement, Human and Health Sciences, University of Rome “Foro Italico”, 00135 Rome, Italy; 5 Department of Human Sciences, Guglielmo Marconi University, Via Plinio 44, 00193 Rome, Italy; 6 Faculty of Medicine, University of Ostrava, 73000 Ostrava, Czech Republic

**Keywords:** angiodysplasia, vascular anomalies, physical activity, Gait Analysis, SF-36 questionnaire

## Abstract

**Background/Objectives**: This case study involved a 24-year-old male with an arteriovenous malformation localized in the long head of the right biceps femoris muscle, with an anterior cruciate ligament injury. The aim was to assess the effects of a five-week kinesiological protocol, which included global postural re-education and strengthening exercises focused on knee stabilization. **Methods:** The effectiveness of the therapeutic intervention was evaluated using Gait Analysis, clinical examination, and the SF-36 questionnaire to assess the patient’s quality of life. **Results:** The study revealed significant postural improvements, including the restoration of the spine’s physiological curves, with kyphosis angles measuring 44.7° in indifferent orthostasis and 41.7° in self-corrected standing; and lumbar lordosis measuring 32.8° in indifferent orthostasis and 41.9° in self-corrected standing. Additionally, there was a restoration of the correct knee, hip, and ankle angles, along with a shift in the principal axis of the center of pressure from 7.6° pre-intervention to 12.9° post-intervention. The patient’s perception of physical efficiency also improved, increasing from 60% to 75% over the treatment period. **Conclusions:** The effectiveness of the kinesiological treatment was confirmed by the improvement in gait stability and overall strengthening. The patient’s active involvement in the treatment process enhanced his confidence in its success, ensuring adherence to the protocols.

## 1. Introduction

Angiodysplasia refers to an abnormal development of blood vessels, which could grow invasively, infiltrating surrounding tissues, and organs, or encapsulating them within proliferative vascular systems. The manifestations of angiodysplasia are highly variable, making each case unique [1]. Vascular anomalies result from errors in the embryogenetic development of vessels and can present at birth, during adolescence, or even in adulthood. Chronic vascular malformations, however, do not regress spontaneously and develop with the individual [2].

To aid in diagnosis and treatment, the International Society for the Study of Vascular Anomalies (ISSVA) has provided an international scientific classification that offers clinicians a simple and practical tool to help specialists select the most appropriate and targeted treatments [3]. According to the ISSVA 2018 classification, at present, hemangioma is diagnosed as an “infantile hemangioma”, categorized under benign vascular tumors. This classification helps differentiate it from other vascular malformations based on its characteristic rapid proliferative phase, followed by a gradual involution [4]. Most cases of arteriovenous malformations (AVMs) involve superficial soft tissues, such as the skin and mucous membranes, but they often extend to deeper planes, affecting muscles, bones, and joints. Infiltrative malformations (extra-troncular) could be classified as focal, localized, or segmental, affecting specific organs or muscle districts. There was a prevalence of these anomalies in the limbs and the cervico-cephalic region [5]. Regarding limbs, AVM varied greatly in size, from localized forms to those involving entire limbs.

The treatment involves complete excision of the AVM nidus and adjacent soft tissues, followed by loco-regional reconstruction with skin or muscle flaps. However, esthetic and functional outcomes are often unsatisfactory, with frequent recurrences. This approach is advisable for circumscribed forms, especially symptomatic intramuscular AVMs [6].

Nowadays, transcatheter embolization of the afferent arteries is the primary approach for treating AVMs. This approach could prevent recurrences due to collateral fistulous circuits and reduce the risk of ischemic damage from accidental embolization of vital organs or tissues [7].

A newer approach involves retrograde treatment of AVMs through sclerotherapy of the draining veins. Sclerotherapy of the dominant draining veins was considered a valuable complement to embolization, particularly when the latter was challenging due to tortuous, small-caliber arteries or those in communication with vital organs [8].

Angiodysplasias could be highly disabling and were managed with delicate surgical interventions that often needed to be repeated over time.

AVMs, depending on their location, size, and associated complications, can lead to various challenges in the patient’s social life, such as chronic pain or fatigue, mobility issues, such as difficulty walking, limiting the ability to participate in social or work activities, and causing social isolation. Consequent mood disorders, such as fear of hemorrhage or worsening of the condition, can result in anxiety and depression, negatively affecting relationships and quality of life [9].

Managing AVMs may require a multidisciplinary approach that includes not only medical and surgical treatments but also physical therapy and psychological support to minimize the impact of disabilities on the patient’s social life.

This study aimed to determine whether a targeted physical activity protocol could support existing therapies and improve the health and well-being of individuals affected by AVMs. Given the rarity of the condition and the lack of specific studies in the literature, this study is a case study.

## 2. Material and Methods

### 2.1. Study Design

The present case study aimed to apply a five-week kinesiological protocol post-chirurgical intervention (from January to November 2020), combining global postural re-education and strengthening intervention for knee stabilization, to the 24-year-old subject affected by MVA, localized in the long head of the right biceps femoris muscle, and with injury of the anterior cruciate ligament.

### 2.2. Case Details

This case involved a 24-year-old male, standing 1.79 m tall and weighing 82 kg. The patient’s lifestyle was highly stressful, involving early mornings and long work hours, and he was involved in amateur sports and playing soccer.

The patient’s medical history includes the diagnosis of an arteriovenous malformation (AVM) localized in the long head of the right biceps femoris muscle. The first symptoms appeared around the age of 14, when he began experiencing persistent pain in his right thigh.

The rehabilitation program was conducted in a step-by-step manner, followed by a structured follow-up approach. This allowed for the necessary time for physiological adaptations, with treatments being adjusted based on the results. The entire process was continuously monitored and assessed through clinical examinations.

The first step was a diagnosis, involving a series of specialist consultations and diagnostic tests, including Doppler ultrasound, MRI, Gait Analysis for postural imbalances, and the Italian version of the SF-36 questionnaire to evaluate the patient’s overall well-being and quality of life.

The second step focused on treating the inflammation caused by the twisting injury through both instrumental (Tecar and magnetotherapy) and manual physiotherapy. After completing physiotherapy, the patient began kinesitherapy, including exercises in closed kinetic chains, and open kinetic chains for strengthening and proprioception.

At this stage, the treatment plan involved surgical intervention for stabilizing the AVM using a sclerosing agent. After the procedure, additional diagnostic tests were performed to confirm the success of the surgery and ensure the patient could safely resume motor rehabilitation.

The motor rehabilitation program began with hydrotherapy sessions in a pool twice a week for about a month, followed by a kinesiology program focused on strengthening and postural re-education.

**Figure 1:**
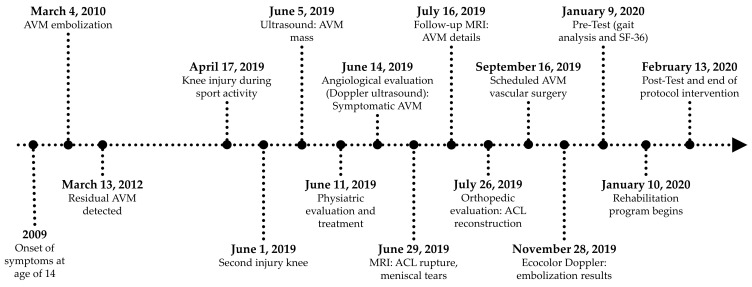
Timeline of the patient’s care, detailing significant dates of surgical interventions, rehabilitation, and follow-up appointments. This timeline illustrates the chronological sequence of important events and procedures related to the patient’s treatment and recovery.

The patient’s medical history is shown in Figure 1.

### 2.3. Assessment

#### 2.3.1. Gait Analysis

The Gait Analysis clinical examination was conducted before and after the postural intervention using the Global Opto-Electronic Approach for Locomotion and Spine (G.O.A.L.S) device manufactured by Bioengineering & Biomedicine Company SRL. This non-invasive test evaluated the 3D morphology of the spine in both static and dynamic conditions, and assessed load distribution, knee flexion-extension, pelvic control, and gait phases. The Gait Analysis was a computerized assessment of gait that recorded, quantified, and tracked changes in gait over time. It is a valuable tool for evaluating patient disorders, monitoring pharmacological, rehabilitative, and re-educational programs, assisting in therapeutic decision-making.

The assessment was performed in an upright stance and self-correction, both statically and dynamically on a treadmill. Thirty-one reflective markers were placed on the subject at predefined reference points. Infrared cameras facilitated the evaluation and study of postural and movement disorders by enabling 3D reconstruction of the whole skeleton in static and/or dynamic conditions. The complete skeletal posture reconstruction was generated by algorithms and highlighted using the specialized G.O.A.L.S. software version 8.6.0.

#### 2.3.2. Questionnaire SF-36

To evaluate the effectiveness of our therapeutic intervention on the patient quality of life, the SF-36 Health Survey was administered [10]. The questionnaire was completed by the patient before, during (after three weeks), and after the rehabilitation process, to monitor health improvements and adjust treatments as needed. The shorter SF-12 version of the questionnaire was used, and it has been validated and translated into Italian [11,12].

### 2.4. Physical Intervention

#### 2.4.1. Intervention for Knee and AVM Stabilization

The rehabilitative intervention for knee stabilization was performed along with postural re-education. The intervention aims to achieve strength of the quadriceps and the hamstrings, the semitendinosus and semimembranosus, and to optimize the residual capabilities of the biceps femoris, which is the site of the arteriovenous malformation (AVM). Moreover, it aims to alleviate post-operative pain and restore proprioceptive systems.

The objective was to prepare the subject for possible arthroscopic knee surgery, with a dry knee, and in a “safe” condition concerning the AVM.

The following exercises were selected for knee rehabilitation: squats, hamstring exercises, shoulder bridges, and exercises on proprioceptive platforms.

#### 2.4.2. Global Postural Re-Education Intervention

The subject underwent targeted postural rebalancing training twice a week, with a focus on strengthening the knee-stabilizing muscles. Specific postural exercises were administered to promote fascial and muscular stretching, aimed at rebalancing muscle chains and eliminating compensations occurring at various levels: pelvis, lower limbs, etc. The training particularly addressed the correction of the extensor muscles of the spine, employing concentric exercises for the iliocostalis and longissimus muscles, to increase lumbar lordosis and reduce kyphotic posture. Special attention was given to diaphragmatic breathing and reinforcement of the core stability.

The exercises were selected to restore mobility and stability to body segments that had lost their efficiency. Exercises were performed in a prone position for the spinal extensors: arms extended and slightly abducted, lifting the torso while maintaining spinal extension, followed by lifting the extended legs, and finally lifting both torso and legs simultaneously and then alternately (Table 1). The workload, in terms of sets, repetitions, and rest periods, varied between sessions to meet the individual needs of the patient. This approach allowed for personalization and optimization of the treatment based on the subject’s response and progress, for both muscle strengthening and posture correction.

## 3. Results

In the initial assessment, sagittal spine alignment was compromised during self-corrected standing, resulting in a nearly straightened spine with 33.5° of dorsal kyphosis and 20.7° of lumbar lordosis. In indifferent orthostasis, the measured angles were 45.9° for kyphosis and 26.8° for lordosis.

Regarding dorsal kyphosis, a comparison of the previously presented graphs indicated that the Cobb angles of the spine were “normalized” following the intervention. Specifically, the kyphosis angles reached physiological values, measuring 44.7° in indifferent orthostasis and 41.7° in self-corrected standing. Additionally, lumbar hypolordosis improved, with the Cobb angle increasing to 32.8° in indifferent orthostasis and 41.9° in self-corrected standing.

**Figure 2:**
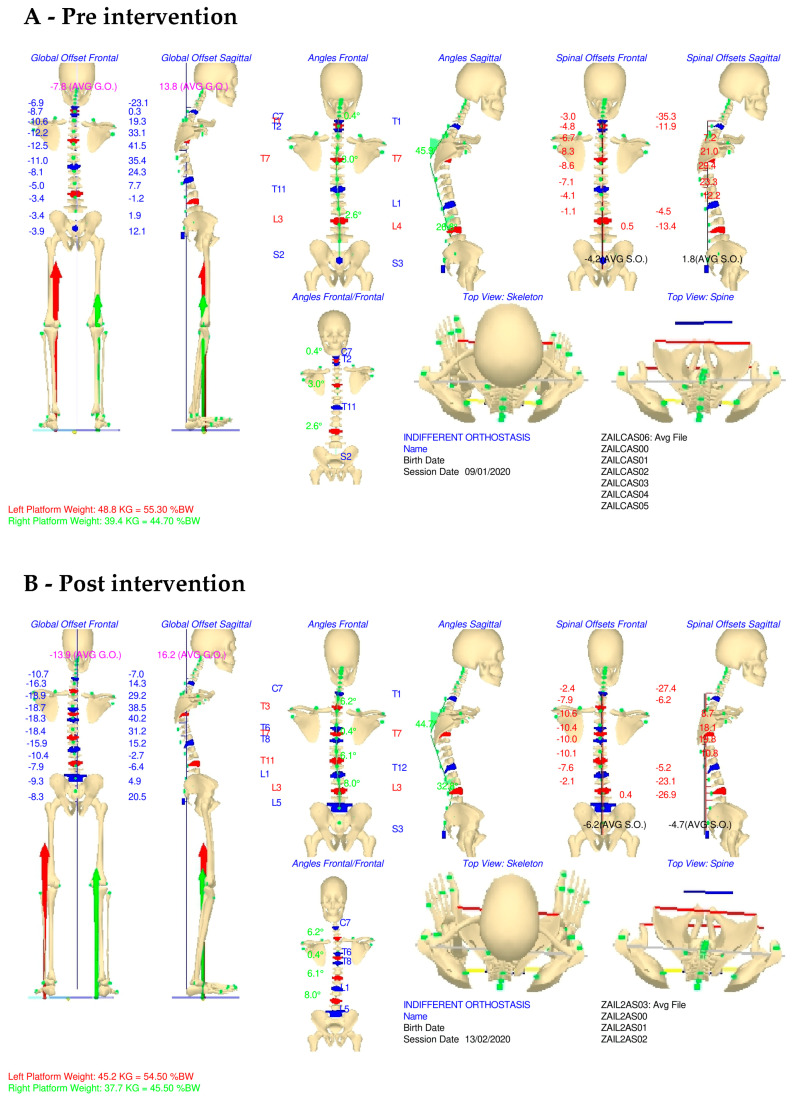
Gait Analysis results before and after intervention.

**Figure 3:**
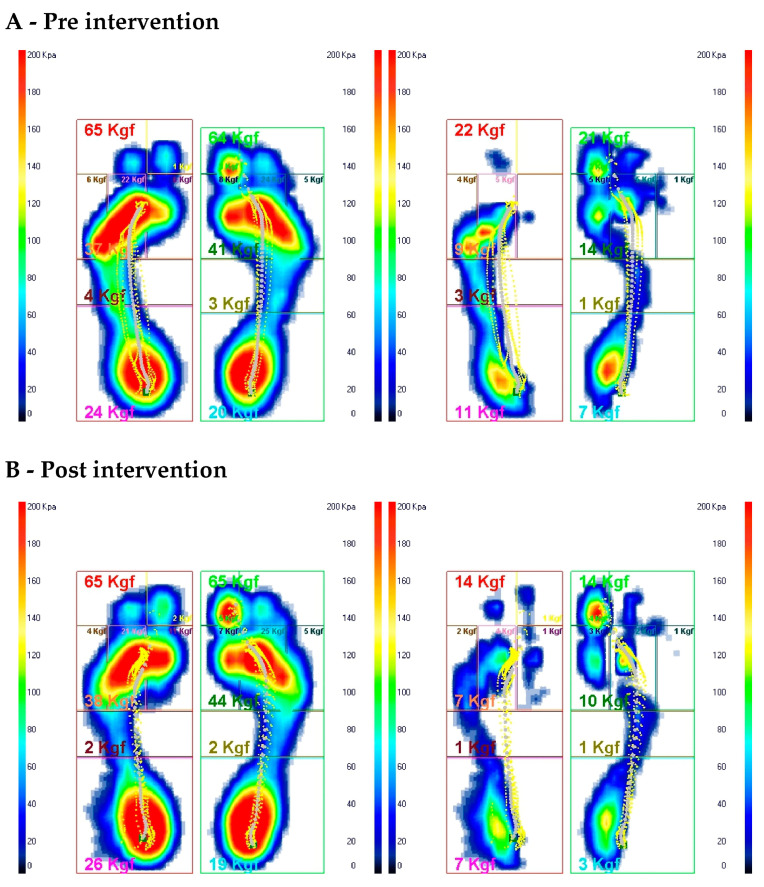
Average gait cycles: distribution of pressures and plantar loads in the peak frame averages and their side-to-side differences before and after intervention.

The principal axis of the center of pressure (COP) support shifted from 7.6° pre-intervention to 12.9° post-intervention. The results of Gait Analysis are shown in Figure 2 and Figure 3.

The restoration of the correct knee angle, hip angle, and ankle angle, following the postural intervention, improved flexion-extension, abduction-adduction, pronation-supination, and internal and external rotation, especially of the right knee affected by ACL injury and AVM, demonstrating enhanced stability [13].

**Figure 4:**
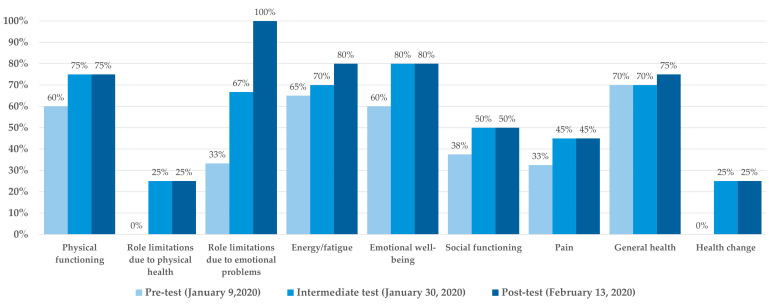
Results of the SF-36 questionnaire in pre-, intermediate-, and post-intervention.

The SF-36 questionnaire revealed an improvement in the subject’s perceived physical efficiency, increasing from 60% to 75% over the course of the treatment. According to the subject’s self-assessment, functional limitations decreased by 25%, and emotional distress issues were fully resolved by the end of the rehabilitation. The score for daily activity well-being increased from 65% to 80%, while emotional well-being improved from 60% to 80%. Social well-being also improved, rising from 37.5% to 50%. Overall, the subject’s perception of his health status showed a significant improvement of 25%. The results of the SF-36 questionnaire are shown in Figure 4.

## 4. Discussion

Gait Analysis, before and after the kinesiological intervention, revealed significant postural improvements within five weeks of treatment.

Improvement of the kyphotic posture and dorsal hyperlordosis, which reached a physiological Cobb angle, respectively, ensuring the effective and efficient restoration of the spine’s physiological curves, is crucial for improving its load-bearing capacity and flexibility [14]. Recent studies have highlighted the importance of core stability and knee proprioception in managing injuries, particularly in patients undergoing ACL rehabilitation, emphasizing the approach used in this study [15,16]. Additionally, the integration of postural exercises aimed at correcting kyphotic and lordotic deviations has been shown to facilitate better load distribution across the lower limbs, which was a critical factor in this case, given the presence of arteriovenous malformation (AVM). According to the collected results, the projection of the COP improved. Post-intervention, the vertical projection of the COP shifted to a position anterior to the lateral malleolus, compared to its previous position toward the rearfoot.

The improved weight distribution in static positions led to more effective load distribution between both limbs which was previously shifted to the left limb. Analysis of the average Gait Cycles, including the regular forces exerted per side and the force trends during the mid-stance phase for each side, showed improvement in the foot, heel, and metatarsal areas. The ACL injury and the pain caused by the AVM significantly altered the patient’s postural alignment; nevertheless, the comprehensive postural rebalancing interventions improved a highly complex postural condition.

The strengthening of the CORE stability improved the pelvic rotation angles, more stable and controlled throughout the gait cycle. The muscles of the hip, knee, and ankle became well-balanced bilaterally [17].

The strengthening of the hamstring chain combined with quadriceps exercises and stretching resulted in improved stabilization of the knee angle, especially compensating for the injury of the anterior cruciate ligament. Furthermore, the recovery of trophic tone in the biceps femoris contributed to a reduction in the pain reported in the AVM region, inducing the subject to remove the graduated compression stocking. The AVM, having been thrombosed and sclerosed, was likely stabilized due to the increased trophic tone and elasticity of the biceps femoris, which provided a ‘containing’ effect. This stabilization improved both the painful symptoms and the sensation of instability.

The SF-36 questionnaire results confirmed the subjective perception of the intervention’s effectiveness. During a rehabilitation period, the perception of well-being and confidence in the rehabilitative treatment enhanced the outcomes and promoted continued engagement in the treatment effectively [18,19].

The subject is likely to approach ACL reconstruction with greater confidence and a higher potential for functional recovery within the biological timeframes.

The protocol was administered to a single subject, as AVM of the biceps femoris is a rare condition that presents with different symptomatology among patients and requires targeted interventions. Furthermore, the case is unique due to the presence of both AVM and an anterior cruciate ligament injury. The combination of postural exercise and muscle strengthening proved to be highly effective. Future studies may identify different combinations of kinesiology treatments based on the clinical presentation and implications of patients with AVM.

## 5. Conclusions

This study was novel and distinctive, as similar interventions for patients with AVM had not been documented in the literature.

It highlighted the effectiveness of the present kinesiology treatment aimed at improving gait stability and general strengthening, actively involving the subject in the implementation of the protocol and keeping him consistently informed about the results. This approach also enhanced his confidence in the success of the treatment. The subject’s emotional and practical engagement ensured adherence to the protocols and led to more significant improvements.

## Figures and Tables

**Table 1:** Summary of Exercises and Their Targeted Outcomes.

Exercise	Purpose
Squats (half and deep)	Strengthening lower limbs, trunk stabilization, activation of quadriceps, hamstrings, and glutes.
Lunges (front, Bulgarian, lateral)	Stimulation of thigh muscles (glutes, hamstrings, quadriceps), sural triceps, adductors, and CORE.
Leg Curl	Strengthening hamstrings, knee stabilization, improving the hamstring: quadriceps ratio (H:Q), specific activation of the biceps femoris muscle.
Shoulder Bridge	Involvement of gluteal and iliotibial muscles, lengthening of the hip flexor compartment.
Exercises on proprioceptive platforms	Rebalancing of the proprioceptive neuromuscular system, increased stability, neuromuscular control, and balance.
Upper body raises (prone position)	Strengthening back extensors.
Plank	CORE activation, strengthening of the transverse abdominis and oblique muscles.
Cobra (prone position)	Extension of the lumbar spine, lengthening of the abdominal muscles and iliopsoas, and flexibility of the lumbosacral hinge.
Posterior kinetic chain stretch	Lengthening of connective tissues of muscles and nerves, lengthening of the spine and posterior muscles of the thigh and leg.
Spine and hamstring stretch	Progressive lengthening of the spine and posterior muscles.
Sitting with back against the wall	Lengthening of hamstrings, glutes, soleus, and gastrocnemius.

## Data Availability

Data is contained within the article.
